# Fabrication of Pillar Shaped Electrode Arrays for Artificial Retinal Implants

**DOI:** 10.3390/s8095845

**Published:** 2008-09-24

**Authors:** Eui Tae Kim, Jong-Mo Seo, Se Joon Woo, Jing Ai Zhou, Hum Chung, Sung June Kim

**Affiliations:** 1 School of Electrical Engineering and Computer Science; E-Mails: arnoldk2@snu.ac.kr (E. T. K.); callme@snu.ac.kr (J. M. S.); sallyjzhou@gmail.com (J. A. Z); 2 Department of Ophthalmology, College of Medicine and Seoul Artificial Eye Center, Nano Bioelectronics & System Research Center (NBS-ERC), Seoul National University, Seoul, Korea; E-Mails: sejoon1@hanmail.net (S. J. W.); hchung@snu.ac.kr (H. C.)

**Keywords:** Polyimide, retinal implant, pillar, optical coherence tomography, electrically evoked cortical potential

## Abstract

Polyimide has been widely applied to neural prosthetic devices, such as the retinal implants, due to its well-known biocompatibility and ability to be micropatterned. However, planar films of polyimide that are typically employed show a limited ability in reducing the distance between electrodes and targeting cell layers, which limits site resolution for effective multi-channel stimulation. In this paper, we report a newly designed device with a pillar structure that more effectively interfaces with the target. Electrode arrays were successfully fabricated and safely implanted inside the rabbit eye in suprachoroidal space. Optical Coherence Tomography (OCT) showed well-preserved pillar structures of the electrode without damage. Bipolar stimulation was applied through paired sites (6:1) and the neural responses were successfully recorded from several regions in the visual cortex. Electrically evoked cortical potential by the pillar electrode array stimulation were compared to visual evoked potential under full-field light stimulation.

## 1 Introduction

Recently, efforts in neural engineering research for vision prosthesis such as retinal implants, optic nerve implants and cortical implants, have increased, with several groups performing clinical trials with the goal of achieving recognition of various objects [1-7].

Visual perception is closely related to the quantity of information, and especially to the spatial resolution. A 0.5 degree of visual angle which corresponds to a visual acuity of 20/700 is considered as the beginning point of the clinically useful visual acuity, thus the primary goal of the retinal prosthesis is targeted to the 0.5 degree of visual angle [8]. According to the minimal requirements for artificial vision in psychophysical studies [9-12], at least 300 pixels should be integrated within about 5 degrees eccentricity. Moreover, for the safe movement of the patient in a dynamic environment, 10-15 degrees eccentricity [9, 10] should be recruited by an additional 200 pixels or so. Therefore, useful vision might be feasible for prosthesis containing more than 500 pixels in 10-15 degrees eccentricity. In the human eye, 3.4 degrees eccentricity is equivalent to 1 mm in diameter on the retina, and as a result, 500 pixels should be integrated into roughly in a 4 mm diameter.

In clinical trials performed to date by several research groups, only low density and comparatively large size of electrode arrays have been applied, such as a 16-channel electrode array in the eye [2] and a 4 to 8 channel cuff-shaped electrode on the optic nerve [1], and several prospective results were reported. Although the resolution of the device is not high, most of patients saw phosphenes and some patients roughly described the position and shape of objects in front of them. Moreover, the recognition time of objects was relatively decreased and converged as the stimulation train continued. Because recognition was performed only at extreme contrast, white and black, and with not enough resolution, these studies do not approximate clinically useful vision.

For more enhanced vision, high resolution systems with well-localized focal point stimulation should be considered. There have been several efforts to make columnar type electrode arrays for retinal prosthesis, including a gold-sited electrode array connected to rigid multiplexer chip [13], a flexible polyimide-based simple post structure [14], a 3D iridium oxide electrode on a polyimide substrate with post and stacked hat structure [15], a convoluted shaped electrode array [16] and a pillar design for optoelectrical stimulation [17]. In this paper, we present a pillar-shaped electrode array for local stimulation to lower the threshold by decreasing the geometrical distance between stimulating sites and target areas.

## 2. Methods

### 2.1. Fabrication of the microelectrode array

**Figure 1.**
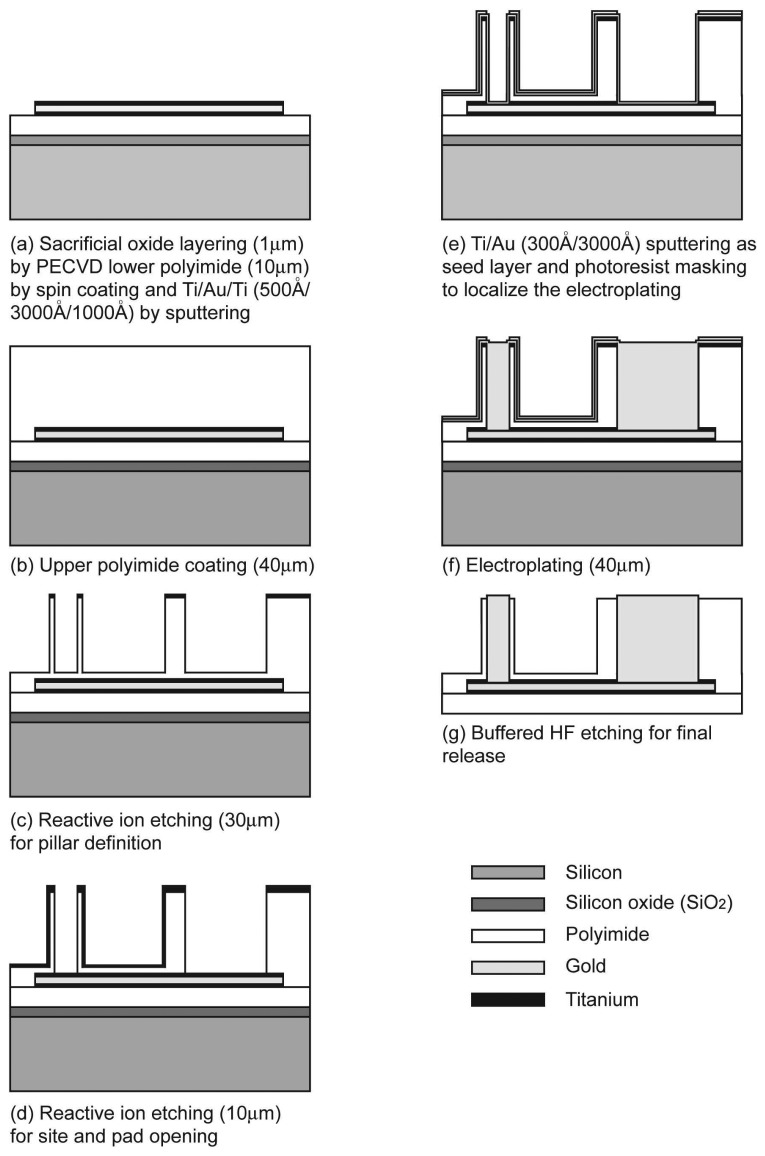
Schematic illustration of the electrode fabrication. (a) Sacrificial oxide layering (1 μm) by PECVD, lower polyimide (10 μm) by spin coating and Ti/Au/Ti (500 Å/3000 Å /1000 Å) by sputtering; (b) Upper polyimide coating (40 μm); (c) Reactive ion etching (30 μm) for pillar definition; (d) Reactive ion etching (10 μm) for site and pad opening; (e) Ti/Au (300 Å/3000 Å) sputtering as seed layer and photoresist masking to localize the electroplating. (f) electroplating (40 μm); (g) buffered HF etching for final release.

The fabrication method was similar to established procedures for the preparation of polyimide-based electrode arrays [18], briefly described in Figure 1. The silicon wafer was pre-deposited with 1 μm silicon oxide by plasma-enhanced chemical vapor deposition (PECVD) as a sacrificial layer, and the polyimide (PI2525, Hitachi DuPont MicroSystem, USA) was spin-coated up to 10 μm in thickness onto the sacrificial layer. Triple layers of titanium (500 Å) - gold (3000 Å) - titanium (1000 Å) were sputtered in order and patterned for metal conduction lines. The lower titanium layer enhanced the adhesion between the polyimide and the gold layer, and the thicker, upper titanium layer protected the gold layer from oxygen plasma attack during site/pad opening.

In order to avoid trapping unwanted air, the upper polyimide layer was coated up to 40 μm in thickness by four repetitive spin-coating and curing procedures. In each repetitive procedure, 10 μm of polyimide was spin-coated and cured on a hotplate up to 150 °C for 20 minutes, except that the final curing was done up to 300 °C for 40 minutes and sustained for 30 minutes. Donut-shape mask patterns were introduced and the two-step reactive ion etching (RIE) was applied for pillar definition and opening the electrode site in the pillar. Another masking and the O_2_ plasma reactive ion etching were done to define the whole electrode shape. To fill the previously defined excavation with gold in donut shaped polyimide columns, titanium (300 Å) and gold (3000 Å) were sputtered as the seed layer, and the photoresist (AZ P4620, AZ electronic materials, Luxembourg) was patterned to localize the electroplating inside the excavation. The gold electroplating was done and the final structure was released by buffered HF etching.

### 2.2. Evaluation of the microelectrode array

The fabricated microelectrode arrays were examined by light and field emission scanning electron microscopy. The impedance and the cyclic voltammetry (CV) of the microelectrode array were tested in phosphate-buffered solution (pH 7.4) and the charge storage was calculated from the CV curve.

New Zealand White Rabbits were used for *in vivo* evaluation of the pillar type polyimide electrode array. All procedures conformed to the Association for Research in Vision and Ophthalmology (ARVO) Statement on Use of Animals in Ophthalmic and Vision Research. The electrode was implanted into a rabbit eye by transscleral suprachoroidal approach. A 4 mm-length scleral incision parallel to the limbus was made with a blade, 4 mm posterior to the limbus at the 1 o'clock site. A 20 μm-thick polyimide guide was used to enhance the insertion of the electrode in the suprachoroidal space and was removed after identifying the proper implantation of the electrode near the visual streak of the rabbit retina. *In vivo* stability of the implanted electrode array was checked by the fundus examination and by the optical coherent tomography (Cirrus OCT, Carl Zeiss, Dublin, CA, USA) [19] for 4 weeks postoperative.

### 2.3. Electrophysiological tests

*In vivo* electrical stimulation was performed to evaluate the functioning of the electrode array in the real situation. Microelectrode arrays were implanted in the right eyes of eight New Zealand white rabbits. Biphasic current pulse with variable pulse amplitude, duration, rate and phase was determined by programmed switches in the custom-made current stimulator. Bipolar electrical stimuli were used to stimulate the rabbit retina with the electrodes paired by surrounding six sites as stimulating electrodes to central one site as a reference [20]. With the stimulation rate of 1 Hz and the duration of 1 msec, the pulse amplitude was gradually increased from 5 μA up to 1 mA by several discrete steps, and the electrically evoked cortical potential (EECP) and the electroretinogram (ERG) were recorded on each amount of amplitude. All the electrophysiological responses were recorded with a multi-channel neurophysiological workstation (Tucker-David Technologies, Alachua, FL, U.S.A.). To analyze the regional responses in the visual cortex according to the electrical stimulation of the retina, EECPs were recorded in twelve spots of the visual cortex. All the spots were located around the area 6 mm to the vertex from the lambdoid suture and 6 mm perpendicular to the right or left [21], and had a 2 mm interval between each other as shown in Figure 5(a). Each recording point was explored with fine dental drill without craniotomy. A disposable single subdermal needle (Rochester Electro-Medical Inc., Tampa, FL, U.S.A.) was inserted about 1.5 μm deep into the hole and the EECP was recorded. The reference electrode was located 20 μm from the lambda to forehead. As a control, the visual evoked potential (VEP) was measured under the full-field light stimulation (EW-202/LS-C, Mayo Corporation, Kyoto, Japan)

## 3. Results and Discussion

### 3.1 Fabrication result

**Figure 2.**
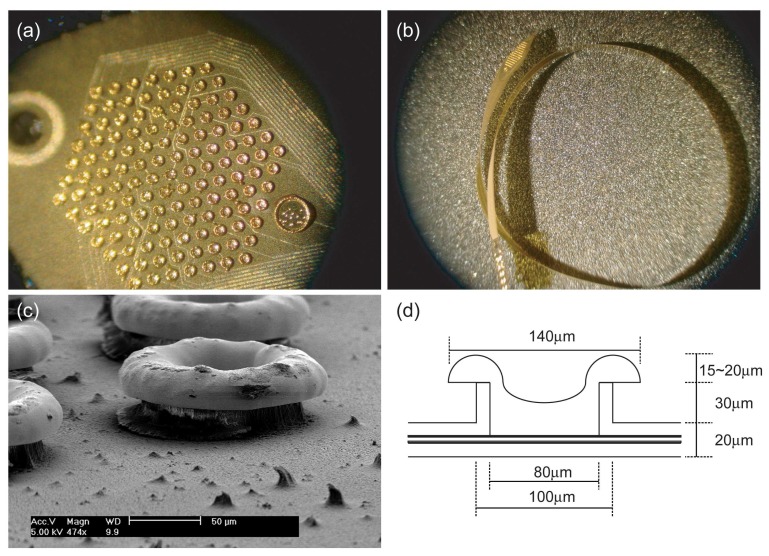
Result of the proposed fabrication process. (a) Electrodes in the stimulation site (top view) and (b) the whole electrode array (side view). (c) Oblique view of pillar electrodes observed with the field emission scanning electron microscope. (d) Diagram of the manufactured pillar electrode.

A pillar-shaped polyimide electrode array was successfully manufactured by the proposed method (Figure 2). Thirty-micrometer-height pillars protruded above the polyimide electrode surface and their tops were covered with mushroom-shaped gold sites, which were unexpected results which added an additional 15-20 μm of pillar height.

### 3.2 Evaluation of the microelectrode array

**Figure 3.**
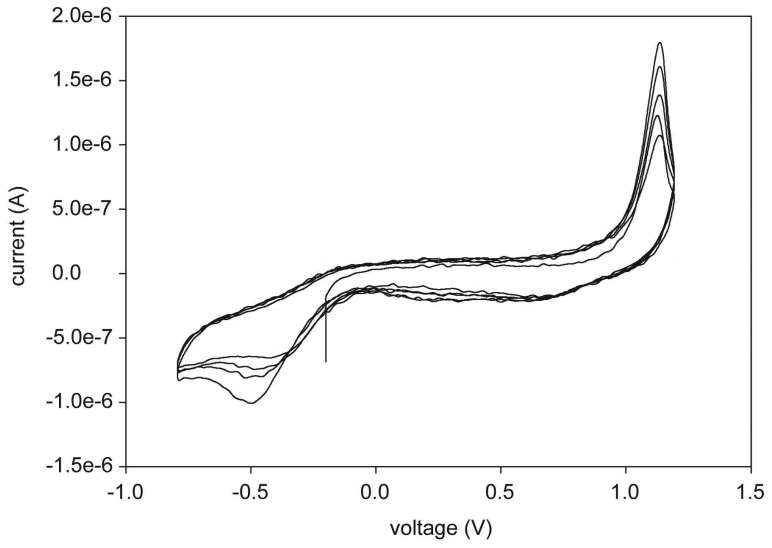
The result of the cyclic voltammetry of the electrode array. The slew rate was 25mV/s and the voltage sweep was from -0.8V to 1.2V in the first 5 cycles.

The impedance of each electrode site was between 10 kΩ to 30 kΩ at 1 kHz. The CV was measured as shown in Figure 3. The positive and the negative peak were found at -0.5 V and 1.1 V respectively on the first path of the CV, and the peak amplitude was gradually increased and converged to -0.8 V and 1.2 V respectively in their direction as the cycle of the CV was repeated. The capacity of the charge storage was calculated from the CV curve by the time integral of the cathodic current during the full potential cycle between the sweep ranges, and was 0.223 mC/cm^2^.

**Figure 4.**
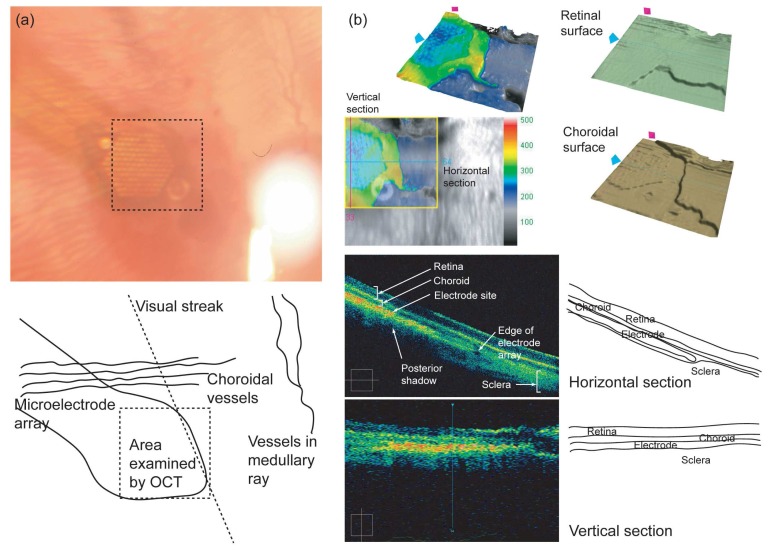
Suprachoroidally implanted microelectrode array in the rabbit eye observed four weeks after implantation. (a) Fundus examination showed that choroidal vessels run over the electrode array and the stimulation site was located near the visual streak. (b) The cross-sectional view (lower half) of the optical coherent tomography revealed that overlying retina and choroids were intact without inflammation or swelling. Indented choroidal surface in the en face view (upper half) reflected the pillar structure of the electrodes.

Fabricated microelectrode arrays were successfully implanted in the rabbit eye (Figure 4). Fundus examination showed that choroidal vessels run over the electrode array and the stimulation site was located near the visual streak. Optical coherent tomography (OCT) revealed that overlying retina and choroids were intact without inflammation or swelling and the microelectrode array was located in the suprachoroidal space in the cross-sectional view. En face view of the retinal surface determined by the inner limiting membrane showed the elevated retina along the suprachoroidally-implanted electrode array.

### 3.3 Electrophysiological test

**Figure 5.**
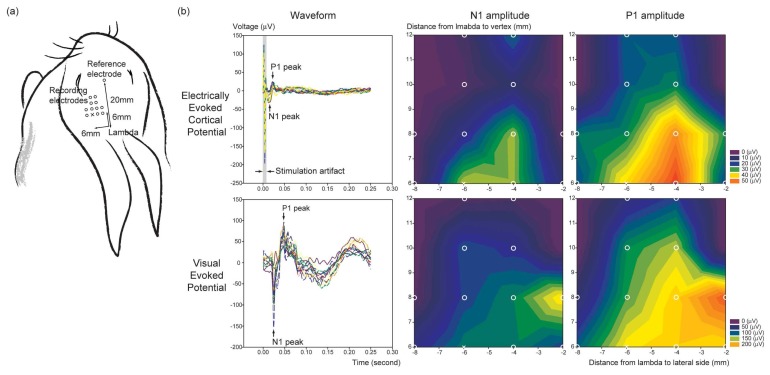
(a) Location of the recording and reference electrodes for the EECP and the VEP measurement in the rabbit. (b) Waveforms of the EECP and the VEP measured by the multi-regional evoked potential recording in twelve spots of the visual cortex, and the isoelectric contour maps of the waveform amplitudes. The EECP and the VEP showed good topographical correlation.

*In* vivo electrical stimulation showed the feasibility of the fabricated microelectrode array in real applications. The EECP was similar to VEP in the shape of waveform, although the electrical retinal stimulation was localized in the posterior pole. The amplitude of EECP was increased according to the increment of the stimulation amplitude. The threshold of the cortical responses to the electrical stimulation of the retina was determined by the discernable N1 and P1 peak on the waveform, and this was turned out to be about 100uA. The multi-regional evoked potential recording in twelve spots of the visual cortex showed good topographical correlation between the EECP and the VEP in terms of N1 and P1 amplitude (Figure 5), although the amplitude map of the VEP showed medially shifted isoelectric contour compared to that of the EECP.

### 3.4 General discussion

The system proposed in this paper has the advantages of simplicity of the fabrication process and pillar shape plasticity. Intuitively, pillar height is determined by coating thickness and the only consideration is that the pillar electrode sites should be filled with gold or another kind of novel material available by electroplating. During the process, electroplating is restricted by aspect ratio between bottom and sidewall height. Because plating occurs both on bottom side of the metal site and the sidewall, an unwanted hollow can be created during the process by closure of the upper part before filling up of the lower part. Though it's not known yet whether this hollow area affects electrical characteristics during electrical stimulation *in-vivo*, more sophisticated electroplating conditions should be used in the future to avoid generating hollows.

In this paper, we fabricated a gold electrode array by electroplating. This was done with a typical setup and charge storage capacity was measured and calculated with a potentiostat (IM6e Impedance Analyzer, Zahner, Germany). Cathodic charge storage capacity (CSC_c_) was shown to be 0.223mC/cm^2^. For sputtered gold in a general setup, it was shown to be 0.239 mC/cm^2^, which is almost similar to the value for electroplated gold. Compared to popular electrode site materials, this is 13 times lower than sputtered iridium oxide (2.8 mC/cm^2^) and similar to titanium nitride (0.25 mC/cm^2^) [22]. Though we have suggested a pillar fabrication process at this time with gold only, our process can be easily extended to other biocompatible metals by additional electroplating with novel materials, such as Pt, and sputtering of iridium oxide, TiN during the procedure.

Electroplating also affects the shape of pillar. In this paper, a mushroom shaped electrode site for retinal prosthesis is reported. It is generated by an edge effect during electroplating. In our study, the mushroom shape was modeled to three half circles on the rooftop of the pillars. A careful field study was not yet done, but the mushroom may contribute to performance in two aspects. One is the shape of surface. For mushroom type arrays, the site has no edge. This means that charges are not driven to spatial regions, site edges, during electrical stimulation. Though the charge storage capacity is the same, spatial dense charges may induce acceleration of site corrosion, so the absence of edges can contribute to the safety of electrode sites of the same type compared to the general edge shaped electrode array. The other is the field overlap. A mushroom shaped sample has a field overlap in the range of each site. Under the same condition, field overlap is more advantageous for enhancing stimulus ability.

For above reasons, we are now studying the field effect of mushrooms according to the reproducible process of mushroom-shaped electrode array fabrication. Though mushroom shapes can be a good solution to reduce an impedance of electrode site for limited surface area, all of sites cannot be strictly controlled due to uncertain factors during the plating sequence. In future work, the relation between the mushroom volume and plating time, including other parameter variations, should be verified for further applications.

The feasibility of the embossed pillar array inside the rabbit eyeball was shown by *in-vivo* OCT. During insertion, the pillar electrode array didn't seem to induce scratches or tissue damage in retina. Wrinkles, which are presumed by slight retinal layer detachment during retinal surgery, were detected in an immediate post-op OCT image. However, tissue layers on the implanted electrode array gradually smoothen after 4 weeks of implantation.

Choroidal vessels run over the electrode array and the fact that the stimulation site was located near the visual streak in the fundus image support the feasibility of the safe implantation of the fabricated electrode array with the proposed surgical technique. The view of the choroidal surface determined by the retinal pigment epithelial layer reflected the pillar structure of the electrodes. This means that the proposed pillar electrode array may deliver the electrical stimuli to the retina more effectively than a planar electrode array does.

For the reconstruction of the cross-sectional view of the OCT, acquired data are calculated under the assumption that reflexes from the inner limiting membrane will form a linear or convex line. As a result, a cross-sectional view cannot visualize the indentations along the choroid by the pillar electrodes in this study. For the same reason, in the horizontal cross-sectional view, a relatively rigid sclera was shown to be reflected backward along the edge of the electrode array even thought the retina and the choroid overlying the edge were visualized as straight lines.

*In-vivo* electrical stimulation and recording was done and compared to a flash LED response. We could not measure repeatability because acute electrical stimulation requires electrode insertion each time before recording procedure, and the electrode site destination was seemed to move slightly. In this paper, one of the recording results was described. However, for whole area light stimulation, it was shown to be repetitive.

In the result, peak response location of pillar electrode array was similar to that of VEP peak. EEP response area was smaller than VEP response area as expected. While, in multi regional EEP response, area broadness of pillar to whole area stimulation by flash LED was not significantly different, peak response amplitude between them had remarkable gap up to 3-5 fold for both the N1 and P1 peaks. Though stimulation threshold can be defined through the EEP response in this setup, EEP peak response discrimination according to locations between channels needs to be more sophisticated in future setup, for example, more dense recording spots. Additionally, the recording electrode insertion setup also needs to be standardized. Recording depth is determined based on the surface of skull to be drilled. However, thickness of skull does not uniform in the whole area; it gets thicker in the direction of the vertex. Angle of insertion is also to be considered. According to the delicate change of drilling direction or electrode insertion direction, target area which is 1.5 μm below to the surface can be significantly changed up to several millimeters. Because skull surface is not flat in ordinary animals, including rabbits, a perpendicular insertion method should be used for more reliable results, especially, for multi-channel recording.

## 4. Conclusions

A pillar type polyimide microelectrode array and its fabrication process are proposed to make an effective stimulation for the artificial retina. Basically, suprachoroidal implant is similar to sub-retinal implant. So our target is to stimulate bipolar/horizontal cells theoretically. However, cell layers to be stimulated are separated from implanting area by the degenerated sparse photoreceptor layer and choroid. We suggest the fabrication process of sidewall insulated pillar to reduce the target distance and enhance the stimulation efficiency. Though we report preliminary result in this time, we expect pillar shape can be an effective solution to realize the further enhanced studies, useful vision.

The suggested procedure is highly flexible for the application of effective stimulation study; controllable height of pillar and variable choice of site material. Because the pillar is made of polyimide with a metal core inside, the metal structure and interfacing tissue can be protected during insertion. Compared to the similar state-of-the-art studies of pillar structures [14, 15], it has an advantage of making a sidewall insulated pillar structure with same kind of insulator, polyimide.

*In-vivo* implantation was successfully performed in rabbit eyes without any damage to the adjacent tissue and showed satisfactory stability for up to four weeks. However, histological study methods should be improved by precision cutting of the sample including sections of pillars in retinal tissue without damage. Bipolar electrical stimulation was done and multiregional recording was established to overcome regional response of the evoked potential which is caused by electrical stimulation or light stimulation.
